# Thermally-Induced Self-Healing Behaviors and Properties of Four Epoxy Coatings with Different Network Architectures

**DOI:** 10.3390/polym9080333

**Published:** 2017-08-02

**Authors:** Liang Fang, Jiamei Chen, Yuting Zou, Zhongzi Xu, Chunhua Lu

**Affiliations:** 1 State Key Laboratory of Materials-Oriented Chemical Engineering, College of Materials Science and Engineering, Nanjing Tech University, Nanjing 210009, China; jiameichen2017@sina.com (J.C.); iceblue546479871@163.com (Y.Z.); zzxu@njtech.edu.cn (Z.X.); 2 Jiangsu Collaborative Innovation Center for Advanced Inorganic Function Composites, Nanjing Tech University, Nanjing 210009, China; 3 Jiangsu National Synergetic Innovation Center for Advanced Materials (SICAM), Nanjing Tech University, Nanjing 210009, China

**Keywords:** self-healing, epoxy coatings, Diels–Alder reactions, reverse plasticity, network architectures

## Abstract

The thermally-induced self-healing behavior of polymer coatings consists of two steps, i.e., gap closure and crack repair. In addition, the polymer coatings with thermally-induced self-healing capability are expected to show satisfied properties to ensure the application. Here, four epoxy coatings with dense irreversible Network I, dense reversible Network II based on a Diels–Alder (DA) reaction, loose irreversible Network III, as well as partially irreversible and partially reversible Network IV were prepared, respectively. The dense irreversible Network I showed an evident gap closure upon heating, while the crack still existed at the high temperature. The dense reversible Network II presented good self-healing upon direct heating at a high temperature of 150 °C, leading to the quick gap closure in 40 s and subsequent crack disappearance in 80 s. The loose irreversible Network III showed negligible crack variations upon heating, while the partially reversible and partially irreversible Network IV showed quick gap closure as well but only partial crack disappearance. Besides, the coating with the reversible Network II based on the DA reaction not only presented good self-healing capability but also possessed the satisfied mechanical properties and the best electrochemical corrosion property, ensuring its further exploitation and potential practical applications.

## 1. Introduction

Since self-healing can prevent economic loss and function failure of polymer coatings as well as increase their lifetime and reliability, self-healing polymer coatings have attracted increasing interest in last decades [1,2,3,4]. External stimulus, usually heat, has been widely utilized to induce the on-demand self-healing of cracks in polymer coatings [5,6]. Similar to the usage of a Band-Aid or suture to close skin wounds before the autonomous healing of tissues, the thermally-induced self-healing of polymer coatings actually consists of two steps as well, i.e., gap closure and crack repair [6]. Only when crack surfaces touch each other tightly, the repair step initiates.

To date, various mechanisms have been explored extensively to deal with the repair step, including molecular re-entanglement [7,8,9,10] and reversible covalent reactions [11,12,13]. For example, fully crosslinked polymers via multi-furan and multi-maleimide groups are prepared via Diels–Alder (DA) reaction [14,15,16,17,18,19,20,21,22]. The retro-DA reaction at temperatures above 120 °C results in the decoupling of reversible crosslinking bonds, while the crack healing is efficiently achieved after the automatic reconnection of the fractured polymer parts via DA reaction at a low temperature. In comparison, the crack closure attracts less attention, even though such a beginning step resulting from surface tension driven viscoelastic flow [12] or reverse plasticity [23,24] was, to some extent, mentioned. In other words, specific chemical structures have been extensively designed for the following repair step, but how these structures affect the initiating gap closure is not widely considered. Such an issue not only limits the understanding of overall self-healing behaviors, but also retards the choice of suitable stimulus procedures to achieve synergetic actions of the close and repair steps.

In addition, it is also significant to evaluate how the basic properties of polymer coatings are affected by the created structures for thermally-induced self-healing. Without satisfied practical performance, the self-healing coatings obviously are of less usability. Therefore, for the purpose of gapping the distance between such advanced function and applications, practical properties of polymer coatings with self-healing capability require investigation.

To address these two issues, we prepared four epoxy coatings with different network architectures in the present work, including dense irreversible Network I, dense reversible Network II based on DA reaction, loose irreversible Network III, as well as partially irreversible and partially reversible Network IV. The aims of this study were, on one hand, to examine the synergetic actions of gap closure and repair involved in thermally-induced self-healing, and on the other hand, to evaluate whether the network architecture based on DA reversible reactions was suitable for creating polymer coatings with sufficient practical properties. The chemical structures of these networks were examined, while their self-healing behaviors under different thermal treatments were observed to clarify how the close and repair steps occurred. Critical coating properties, including UV aging resistance, mechanical properties, and anti-corrosion properties were studied as well.

## 2. Materials and Methods

### 2.1. Materials

**Scheme 1:**
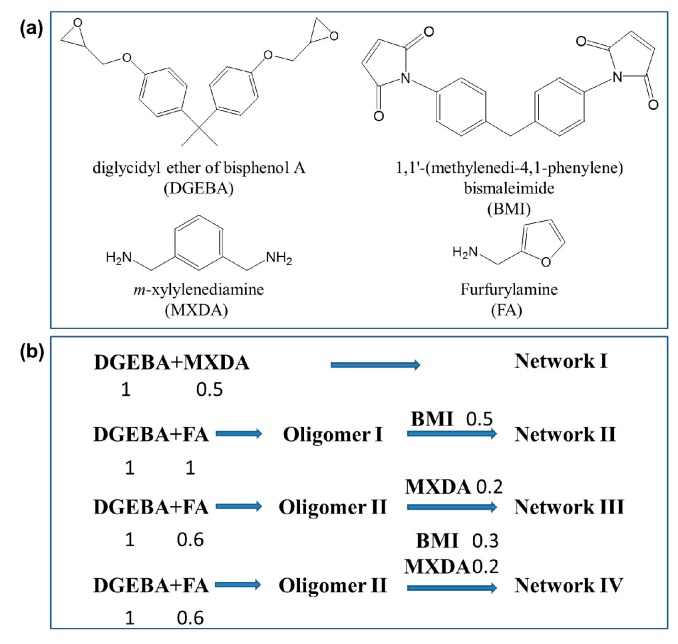
(**a**) Chemical structures of the used reagents; (**b**) Synthesis procedures of Network I–IV, while the number stands for the relative mole number of the chemicals used in each step.

Diglycidylether of bisphenol A (DGEBA), *m*-xylylenediamine (MXDA), and furfurylamine (FA) were all obtained from Sigma Aldrich (St. Louis, MO, USA). Bis(4-maleimidophenyl)methane (BMI) was achieved from J & K Chemical (Beijing, China). Their chemical structures are shown in Scheme 1a. *N*,*N*-dimethylformamide (DMF) was purchased from Aladdin (Shanghai, China). All chemicals were used without further purification.

### 2.2. Sample Preparation

Epoxy coatings with four different networks were prepared according to the procedures described in Scheme 1b.

The epoxy coatings with dense irreversible Network I were synthesized via the reaction of DGEBA and MXDA in a stoichiometric ratio. DGEBA of 1 g (0.00294 mol, containing 0.00587 mol epoxy groups) was added into a glass vial and heated to 100 °C before MXDA (0.2 g, 0.00147 mol, containing 0.00294 mol NH_2_ groups and 0.00587 mol reactive hydrogens) was added, while the mixture was stirred vigorously by hand for several times. Spin coating technology was used to create the epoxy coating on glass slides or tinplates. A certain amount of DMF was added to adjust the mixture viscosity and as a result the film thickness. The mixture was carefully poured onto the plates located on the sample holder of a spin coater (KW-4A, Institute of Microelectronics of Chinese Academy of Science, Beijing, China), while the spin coating was performed at 700 rpm for 1 min. The curing was performed at 60 °C for 2 h in a vacuum oven, 100 °C for 3 h, and 140 °C for another hour. The bulk samples were prepared via pouring the mixture without DMF into a Teflon mold, and the same curing procedure was performed.

For preparation of the coatings with dense reversible Network II based on the DA reaction, loose irreversible Network III, as well as partially irreversible and partially reversible Network IV, Oligomer I and II were synthesized first via the reaction of DGEBA and FA. The molar ratios of DGEBA and FA were 1:1 (stoichiometric ratio) and 1:0.6 (non-stoichiometric ratio) for Oligomer I and Oligomer II, respectively. DGEBA of 24 g (0.0705 mol, containing 0.141 mol epoxy groups) and FA of 6.848 g (0.0705 mol, containing 0.0705 mol NH_2_ groups and 0.141 mol reactive hydrogens) for Oligomer I or of 4.108 g (0.0423 mol, containing 0.0423 mol NH_2_ groups and 0.0846 mol reactive hydrogens) or Oligomer II were dissolved in DMF (40 g) in a sealed conical flask. The solution was heated quickly to 100 °C using an oil bath and 6 h was allowed for the reaction. The oligomers remained in DMF without precipitation, and the weight ratios, thus, were 43.5 wt % and 41.3 wt %, separately. 

The epoxy coating with the dense reversible Network II was synthesized via the DA reaction among the functional furan groups from Oligomer I and maleimides from BMI. The Oligomer I/DMF solution (2.95 g, containing 1 g DGEBA and 0.285 FA, i.e., 0.00293 mol dangling furan groups) and BMI (0.526 g, 0.00147 mol, containing 0.00293 mol maleimide groups) were mixed in a glass vial. The molar ratio of furan and maleimide groups was 1:1 to expect the formation of the dense reversible Network II. The same spin coating procedure was performed on either glass slides or tinplates, while the specimens were cured at 60 °C in a vacuum oven for 12 h. The mixture containing DMF was poured into a Teflon mold as well and allowed 7 days for DMF evaporation naturally, before the treatment at 60 °C in a vacuum oven for 12 h. 

The epoxy coating with the loose irreversible Network III was synthesized with Oligomer II/DMF solution (2.84 g, containing 1 g DGEBA and 0.171 g FA, i.e., 0.00235 mol unreactive epoxy gropus and 0.00176 mol furan groups) and MXDA (0.08 g, 0.00059 mol, containing 0.00235 mol reactive hydrogens). Here, the molar ratio of the theoretically remained epoxy groups in Oligomer II and the amine groups in MXDA was 2:1. The preparation methods for the coatings and the bulk materials were consistent with Network II. 

In addition to MXDA (0.08 g, 0.00059 mol, containing 0.00234 mol reactive hydrogens), BMI of 0.316 g (0.00088 mol, containing 0.00176 mol maleimide groups) was added together into Oligomer II/DMF solution (2.84 g, containing 1 g DGEBA and 0.171 g FA, i.e., 0.00235 mol unreactive epoxy groups and 0.00176 mol furan groups) to prepare the epoxy coating with the partially reversible and partially irreversible Network IV. The roles of BMI and MXDA were to consume the furan groups and the remaining epoxy groups on Oligomer II chains. The preparation methods for the coatings and the bulk materials were consistent with Network II and III.

### 2.3. Crack Formation

A scalpel was attached on the head of a Pencil Hardness Tester (H501-1, Elcometer, Manchester, UK) to create fresh cracks on coating surfaces. The sharp scalpel cut the coatings via moving the head along the coating surface slowly.

### 2.4. Characterizations

#### 2.4.1. Fourier Transform Infrared Spectroscopy

The chemical structures of the prepared two oligomers and four epoxy resins after reactions in Teflon molds, as well as the reversibility of DA and retro-DA reactions in Network II were determined using Fourier Transform Infrared Spectroscopy (FTIR, Vector 22, Bruker, DeKalb, IL, USA). The bulk samples were ground with potassium bromide (KBr) and pressed into testing tablets.

#### 2.4.2. Swelling Experiments

The crosslinking density of the epoxy networks was assessed by swelling experiments. One set of the samples (Network I–IV) was stored in DMF, respectively, for 24 h to evaluate the gel content and swelling ratio. The original mass of the sample was recorded as *m*_1_. After removing from DMF after 24 h, the sample mass (*m*_2_) was immediately measured in the swollen state after cleaning the extra solvent with tissue. The swollen sample was heated at 60 °C until negligible weight variation was observed, before the final mass (*m*_3_) was measured. 

The gel content (*G*) was given by *m*_3_/*m*_1_ × 100%. 

The swelling degree (*Q*) was calculated using Equation (1). The *ρ*_1_ and *ρ*_2_ were the specific densities of DMF and DGEBA.

(1) $$Q=\left[1+\rho_{2}\left(\frac{m_{2}}{\rho_{1}m_{1}}-\frac{1}{\rho_{1}}\right)\right]\times 100\%$$

#### 2.4.3. ^1^H-NMR Analysis

The chemical structures of Oligomer I and II were investigated using ^1^H-NMR (DRX500, Bruck, Germany). The oligomers were completely dried at 60 °C before testing. A small amount of the oligomers were dissolved in the deuterated reagent (DMSO) for scanning.

#### 2.4.4. Gel Permeation Chromatography

Gel Permeation Chromatography (GPC, 515-2410, Waters, Milford, MA, USA) was used to determine the molecular weight of the synthesized oligomers, which were fully dissolved in tetrahydrofuran as the solvent. The flow rate was 1.0 mL min^−1^, while monodispersed polystyrene was used as the standard sample.

#### 2.4.5. Thermal Property Analysis

The thermal behaviors of the four networks were characterized using differential scanning calorimetry (DSC, 204 Phoenix, Netzsch, Selb, Germany). The scanning temperature ranged from room temperature to 150 °C, before it was cooled to −75 °C and then increased to 150 °C again. The heating and cooling rates were 10 °C min^−1^. Degradation temperature (*T*_d_) of the four networks were determined using thermogravimetric analysis (TGA, Netzsch SAT 449C, Selb, Germany) in an air flow with increasing the temperature from room temperature to 800 °C at the heating rate of 10 °C min^−1^.

#### 2.4.6. Morphology Characterization

Optical microscopy (BA210, Motic China Group Co., Ltd., Xiamen, China) was used to evaluate the self-healing behaviors of the four epoxy coatings on glass plates upon varied thermal treatments.

#### 2.4.7. Coating Mechanical Properties

The mechanical properties of the epoxy coatings on tinplates were assessed according to the standard test methods: pencil hardness GB/T 6739-96, adhesion GB/T 1720-89, and flexibility GB/T 1731-93 [25].

#### 2.4.8. UV Aging Resistance

The UV accelerating experiments were performed using an Accelerated Weathering Tester (QUV/se, Q-Lab, Westlake, OH, USA). The UV light irradiation (UVB) power density was 0.61 W m^−2^ and the temperature was 47 °C. The color difference and yellowness index were calculated from the reflection values of the coatings after UV aging for a certain number of days using a UV–Vis spectrometer (UV-3101PC, Shimadzu Co., Tokyo, Japan). The calculation was performed using a home-made computational procedure based on the methods described in a previous report [26].

#### 2.4.9. Corrosion Resistance

Tafel analysis was carried out in a 3.5% NaCl solution on coated steel surfaces using a Potentiostat (CHI66D, Chenhua Instruments Co., Shanghai, China). The area of 1 cm^2^ was used for testing. The coated sample, Pt, and SCE were used as working, counter, and reference electrodes, respectively. The scan range was between −250 mV and 250 mV at a scan rate of 1 mV s^−1^. To assess the self-healing efficiency, the coated plates with a fresh crack and a healed crack at 150 °C for 10 min were immersed in a 3.5% NaCl solution for a certain number of days, while the corrosion situations were recorded.

## 3. Results and Discussion

**Scheme 2:**
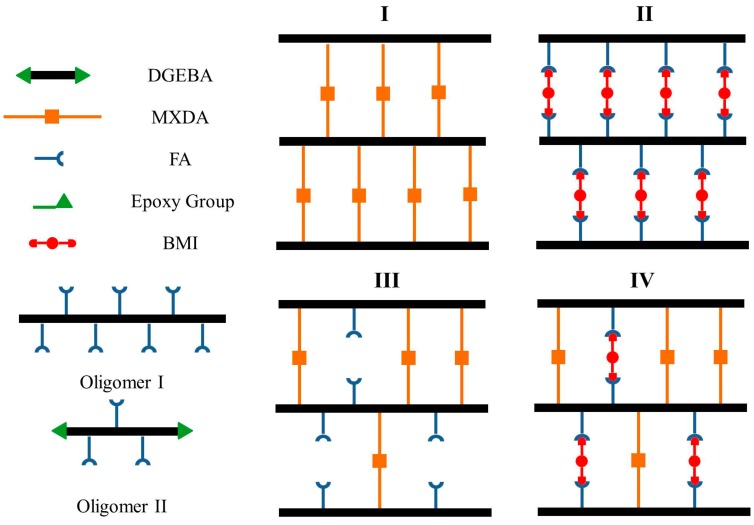
Schematic diagrams of the chemical structures of Networks I–IV and Oligomers I–II.

The dense irreversible epoxy Network I (Scheme 2), which is usually involved in practical applications, was first prepared as the basic architecture based on the curing reaction of DGEBA and MXDA in a stoichiometric proportion. Because of its relatively fast kinetics and mild reaction conditions, the DA reversible reaction was utilized to create the dense reversible Network II as shown in Scheme 2. DGEBA was reacted with FA in a stoichiometric proportion to synthesize Oligomer I first, and the reversible crosslinking was then achieved via the DA reaction among Oligomer I and BMI. As can be seen in the third architecture, a loose irreversible Network III was obtained via the curing reaction between MXDA and the remained epoxy groups in Oligomer II which was synthesized from DGEBA and FA in a non-stoichiometric proportion. The molar ratio of DGEBA to FA in Oligomer II was 1:0.6, i.e., the molar ratio of epoxy/amine groups was 3.333. The partially reversible and partially irreversible Network IV was finally generated. In addition to the irreversible connection resulting from MXDA, BMI was introduced to Oligomer II simultaneously to create the reversible coupling.

The chemical structures and thermal properties of Oligomer I and II were investigated using FTIR, ^1^H-NMR, GPC, and DSC. The four network architectures were examined subsequently using FTIR, DSC, TGA, and swelling experiments. How thermally-induced self-healing of the coatings with different architectures behaved was evaluated based on the observation of the closure and repair steps. Mechanical properties, UV aging resistance, and corrosion resistance of the four coatings were also characterized.

### 3.1. Structures and Properties of Oligomers

The reactivity of DGEBA with FA has been reported in previous reports, facilitating the determination of synthesis conditions for the oligomers [17,18,27]. As reported, the reaction among DGEBA and FA initiated at 57 °C with a maximum exothermal peak at 117 °C [27]. A significant portion of FA (b.p. 147 °C), however, volatilized at 105 °C. In addition, FA was considered to provide a reaction between labile hydrogens in furan rings with epoxy groups, contributing to an irreversible network [27]. Besides, the polymer chains with moderate molecular weight and correspondingly good mobility are commonly required for healing [10]. For these reasons above, the reaction temperature and time were chosen as 100 °C and 6 h in this case.

**Figure 1:**
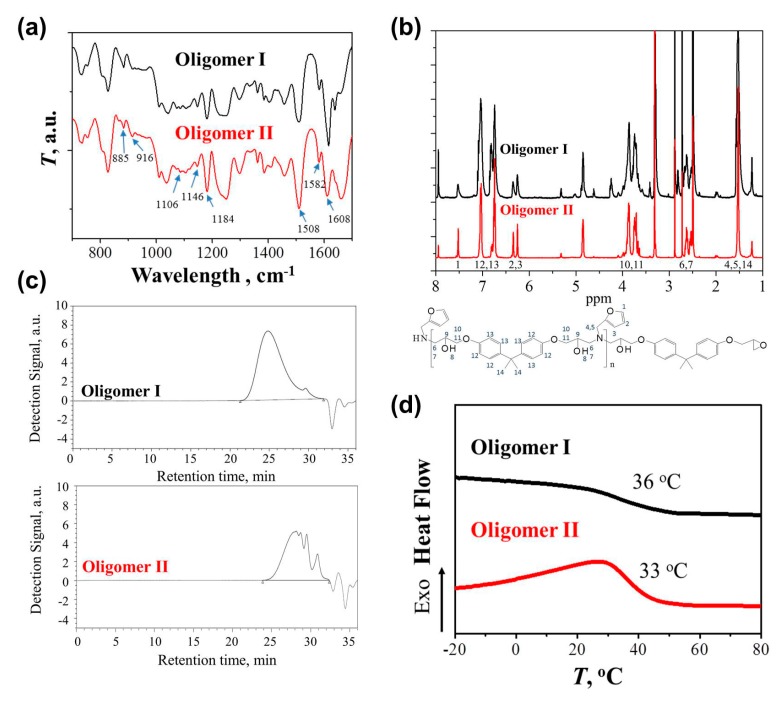
(**a**) Fourier transform infrared spectroscopy (FTIR) spectra; (**b**) ^1^H-NMR spectra; (**c**) Gel permeation chromatography (GPC) plots; and (**d**) Differential scanning calorimetry (DSC) curves of Oligomers I and II.

The chemical structures of the synthesized Oligomer I and II were characterized by FTIR first. As shown in Figure 1a, the peaks at 1508, 1582, and 1608 cm^−1^ were attributed to the C=C bonds of the 1,4 substituted benzene from DGEBA, while the peak at 1184 cm^−1^ was assigned to the benzenic C-H bonds [28]. Because a stoichiometric proportion of epoxy and amine was used in Oligomer I, neglected epoxy groups from DGEBA were determined from the peak at 916 cm^−1^, which was related to the epoxide rings [28,29,30,31]. In comparison, unreacted epoxy groups were found in Oligomer II, providing the reactivity points for further curing. The incorporation of the FA moiety generated the peaks belonging to furan rings, including those at 885, 1106, and 1146 cm^−1^ [15,16,19,20,21]. The results from the ^1^H NMR spectra were also used to confirm the successful synthesis (Figure 1b). The spectra of the two oligomers were the superposition of signals from DGEBA and FA [17,20,22]. The peaks at 7.56, 6.35, and 6.26 ppm were contributed by protons 1, 2, 3 of furan rings resulting from FA, while the DGEBA moiety was responsible for the typical peaks at 6.74–7.11 ppm, corresponding to the aromatic protons 12, 13 [17,20,22]. The peak at 1.6 ppm was contributed by methylene protons 4, 5 from FA as well as methyl protons 14 from DGEBA [17,22].

The molecular weight of the synthesized oligomers was measured by GPC (Figure 1c). Oligomer I exhibited the *M*_w_ and *M*_n_ of 17.2 and 5.9 kDa, respectively, with a PDI of 2.9. Several overlapping peaks were observed in Oligomer II with the non-stoichiometric proportion, corresponding to *M*_w_s of 4.4, 1.6, 1.0, and 0.5 kDa, respectively, while the PDI varied from 1.1 to 1.3. Several research groups have also reported the molecular weight of DGEBA-FA oligomers. Palmese et al. performed the reaction of the stoichiometric reagents in 15 wt % DMF at 90 °C for 30 h [18]. The achieved oligomer presented a *M*_n_ of 4.2 kDa. Turkenburg and coworkers synthesized the oligomer in a non-stoichiometric ratio (epoxy/amine = 2.5) at 125 °C for 2 h, which exhibited the *M*_n_ and PDI of 1.3 kDa and 2.9 [17]. Besides, Mignard et al. prepared the oligomers containing different amount of FA moieties via the condensation reaction among DGEBA, FA, and phenyl glycidyl ether as the end-capping reagent (epoxy/amine = 2) at 100 °C for 15 min [16]. With the aid of catalyst, the *M*_n_s for deca-, hexa-, and tetra-furan oligomers were 4.1, 2.5, and 1.6 kDa, respectively. The molecular weights of the oligomers synthesized in the present work were relatively higher in both stoichiometric and non-stoichiometric ratios, possibly because of the high reaction temperature or the long synthesis time. The glass transition temperatures (*T*_g_) of the oligomers were measured using DSC. As shown in Figure 1d, the *T*_g_s of Oligomer I and II were 36 and 33 °C, respectively. As reported by Turkenburg and coworkers, the *T*_g_ of the oligomer with a relatively small *M*_n_ (1.3 kDa) was 41 °C [17], while the deca-, hexa-, and tetra-furan oligomers, reported by Mignard et al., presented the *T*_g_s of 34, 34, and 20 °C, respectively [16]. These results suggest that the dependence of *T*_g_ on oligomer *M*_n_ was not strong. It might be PDI that played a more dominant role.

### 3.2. Structures and Thermal Properties of the Four Networks

**Figure 2:**
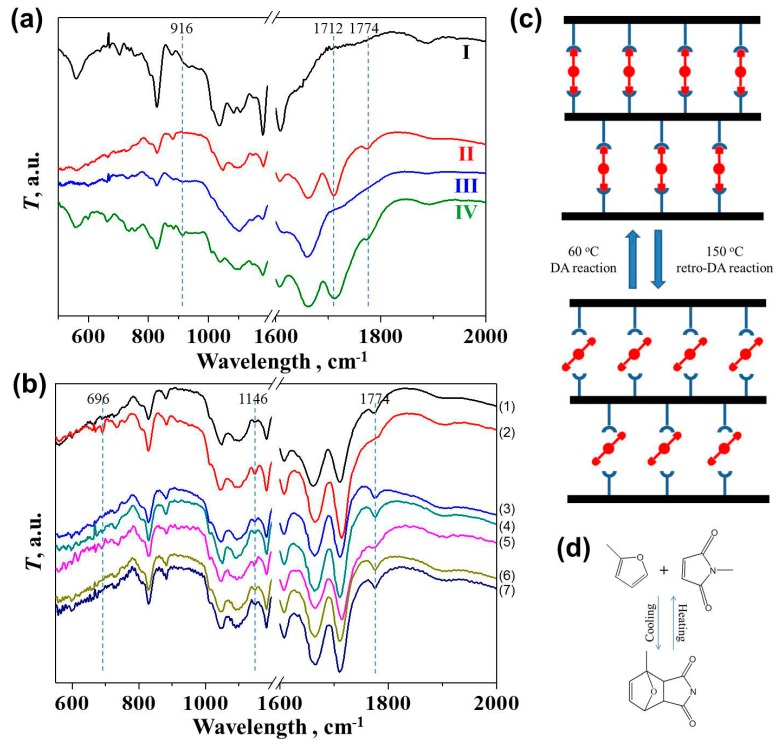
(**a**) FTIR spectra of Networks I, II, III, and IV; (**b**) The FTIR spectra evolution of Network II after different thermal treatments: (1) as prepared, (2) 150 °C for 10 min, (3) 60 °C for 1 h, (4) 60 °C for another hour, (5) 150 °C for 10 min, (6) 60 °C for 1 h, (7) 60 °C for another hour; (**c**,**d**) Schematic diagrams showing the reversibility of Network II and the DA reaction.

After synthesis of the oligomers, four epoxy materials with different networks were created according to Scheme 1. As shown in the FTIR spectra (Figure 2a), no evident peak was observed at 916 cm^−1^ for dense irreversible Network I and loose irreversible Network III, indicating the complete curing of unreacted epoxy groups with the amine groups from MXDA [28,29,30,31]. The curing conditions for the dense irreversible Network I were 60 °C for 2 h, 100 °C for 3 h, and 140 °C for another hour, as presented previously [9,32]. We have shown in a previous report that a sufficient conversion of epoxy groups over 93% occurred within the first hour of the reaction between DGEBA and MXDA at 60 °C [32]. Therefore, the synthesis condition for Network III here (60 °C for 12 h) was also considered to ensure the complete curing reaction. A tiny peak was observed in the partially reversible and partially irreversible Network IV. Under the same curing condition as Networks II and III, some epoxy groups remained in Network IV possibly because the DA reaction retarded the curing reaction via increasing the viscosity. Due to the involvement of the DA reaction, the curing temperature cannot be set over 60 °C. With BMI participation, two evidently new peaks at 1712 and 1774 cm^−1^ were found in Network II and Network IV [21,33,34]. The peak at 1712 cm^−1^ presented the C=O bond in maleimides, while the peak at 1774 cm^−1^ typically presented the existence of DA adducts, suggesting successful DA reactions [21,33,34].

An off-site FTIR experiment (Figure 2b) was performed to examine the reversibility of the crosslinking bonds (Figure 2c) resulting from the DA-rDA reaction (Figure 2d) in Network II. Such variation in the spectra of Network IV cannot be distinguished clearly because less BMI was introduced. The heating and cooling conditions were selected based on the previous full investigation on the thermodynamic and kinetic factors in the DA reaction to ensure the decoupling and recoupling of the fractured polymeric parts [15,21,22]. After heating the sample at 150 °C for 10 min, the distinguishing peak at 1774 cm^−1^ became less evident, indicating the disappearance of DA adducts and the occurrence of the retro-DA reaction [22]. The DA reaction took place again during the subsequent treatment at 60 °C for 1 and 2 h, leading to the peak’s reappearance. Such variation repeated in the second heating and cooling cycle. In addition, the peak at 696 cm^−1^ attributed to the maleimide characteristic band showed up at the high temperature, before it vanished again at 60 °C [21]. Similar repeated change in peak intensity can also be observed for the peak at 1146 cm^−1^, corresponding to furan rings which were consumed by BMI at 60 °C but reappeared at 150 °C [22].

**Figure 3:**
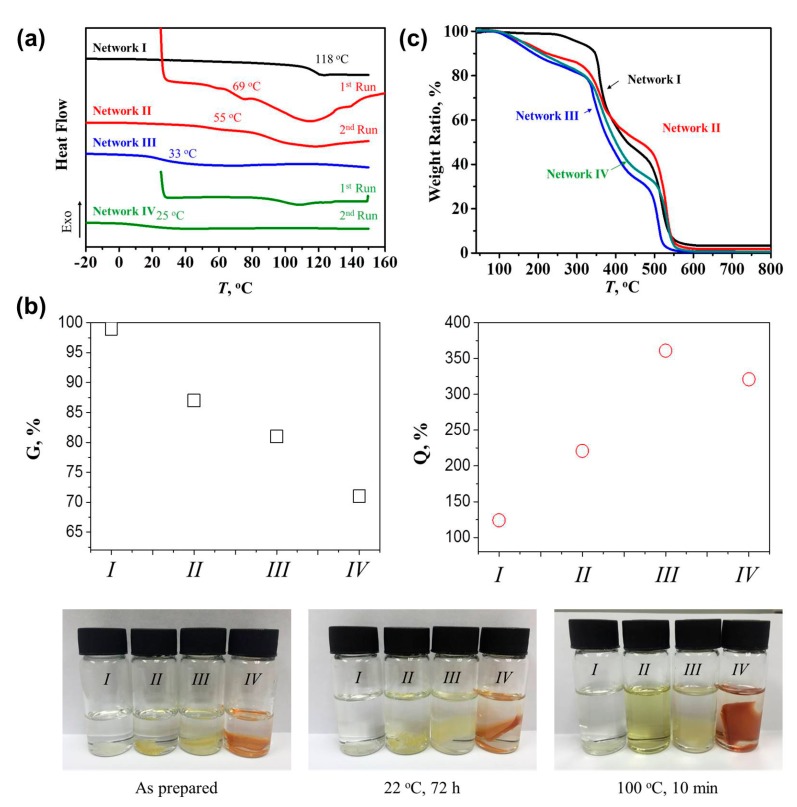
(**a**) DSC curves of the four networks after different heating runs; (**b**) measured or calculated gel content, swelling ratio, and crosslink density of the four networks, as well as images of the solubility of such networks in DMF after different treatment; (**c**) TGA curves of Network I to IV in an air flow.

As shown in Figure 3a and Table 1, Network I presented a *T*_g_ of 118 °C, close to a previous report [35]. Network III showed a *T*_g_ of 33 °C, similar to that of Oligomer II, suggesting that the introduction of a loose crosslinking network through the reaction of the remaining epoxy groups in Oligomer II with MXDA caused negligible influence on *T*_g_. The thermal reversibility of Network II and IV can also be demonstrated via DSC analysis. Network II exhibited a *T*_g_ of 69 °C because the sufficiently generated reversible DA adducts contributed to the dense crosslinking network and the limitation of chain mobility in Oligomer I (*T*_g_ = 37 °C). A following endothermic peak from 80 to 130 °C was assigned to the retro-DA reaction with an enthalpy of 1.59 J g^−1^ [20,21]. The DA reaction occurred again during the subsequent cooling step, leading to the regeneration of DA adducts and correspondingly another endothermic peak with a smaller enthalpy of 1.04 J g^−1^ in the second heating treatment. The reduction in the enthalpy suggested that the relatively fast cooling procedure did not allow the complete DA reaction. The *T*_g_, thus, decreased to 55 °C, while the unconnected BMI may act as plasticizer. Network IV also exhibited an endothermic peak of 1.01 J g^−1^ since less DA adducts contributed to the network. Interestingly, the ratio of rDA reaction enthalpies in Network II and IV was 1.58, close to the theoretical ratio (1.67) of the incorporated BMI amount. During the second heating, no evident endothermic peak was observed. We argue that the regeneration of DA adducts became negligible, possibly because of the existence of irreversible crosslinking bonds. The unreacted BMI thus reduced the *T*_g_ as the plasticizer to 25 °C, lower than Oligomer II and Network III.

The swelling degree and crosslinking density of the four networks were determined subsequently using swelling experiments (Figure 3b). As expected, Network I and III presented the gel content (*G*) of 99% and 81% as well as the swelling ratio of 124% and 361%, respectively. Network II exhibited the *G* and *Q* of 87% and 221%, suggesting a relatively looser network than the dense irreversible Network I. As reported [19], at 60 °C, the conversion between furfuryl alcohol and BMI increased slowly towards 65% within the first 5 h, at which the maximum conversion was almost reached. Due to the steric hindrance of polymer chains, the conversion between furan-ring-terminated oligomers and BMI was even lower [19]. Therefore, in our case, the amount of reversible crosslinks (Network II) based on DA reactions cannot be comparable to the irreversible crosslinks (Network I), generating a lower crosslinking density. Besides, it is interesting to observe that Network IV possessed a lower G of 71% than Network III (81%), indicating that the DA reaction may retard the reaction among epoxy groups and amines as well as the formation of a dense network. The thermo-reversibility of the DA reaction was confirmed by the complete dissolution of the Network II in DMF at 100 °C for 10 min. The other three networks which contained the irreversible network remained insoluble.

The thermal resistance of the four networks was evaluated using TGA (Figure 3c). After dehydrogenation, the decomposition of epoxy resins consists of two steps: decomposition into stable carbonaceous char and volatiles as well as the degradation of carbonaceous char into volatiles [36]. As shown in Table 1, Network I presented the best thermal resistance during the first step, i.e., the temperature at 5% weight loss was 305 °C, while Networks II, III, and IV presented the specific temperature values of 164, 144, and 160 °C, respectively. The loss in crosslink density may contribute to such reduction. The introduction of FA and BMI into epoxy networks increased the degradation temperature within the second step, i.e., the temperatures at the 50% weight loss of Network II and IV were 462 and 400 °C, higher than those of Network I (429 °C) and III (383 °C), respectively.

### 3.3. Thermally Induced Self-Healing Behaviors of the Four Coatings

After the preparation of four epoxy coatings with different network architectures, their thermally-induced self-healing behaviors were investigated to clarify how the close and repair steps occurred. Carbon black (0.5 phr) was introduced as the trace substance to ensure the comparison of the same area before and after thermal treatment, while a similar crack width of 20–30 μm was created on all the four coatings.

**Figure 4:**
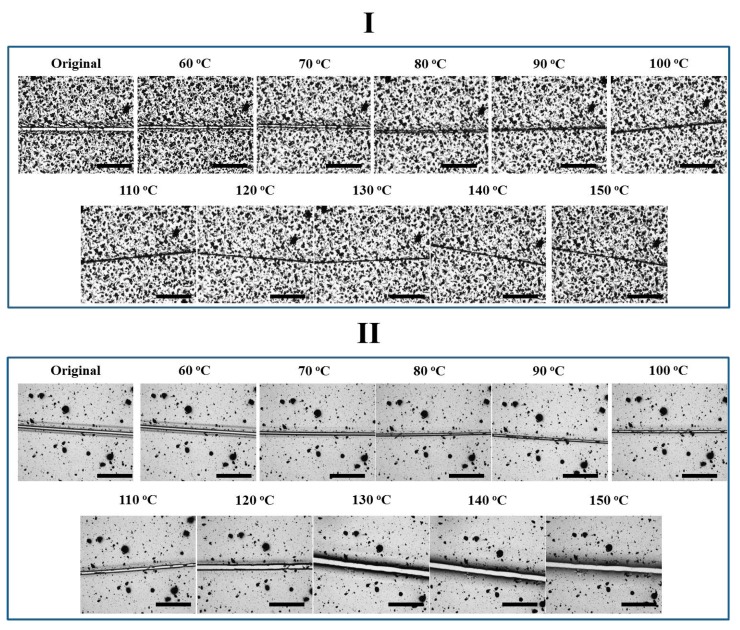
Optical images of the coatings with Networks I–II under thermal treatment of different temperatures for 10 min each. The scale bar stands for 300 μm.

**Figure 5:**
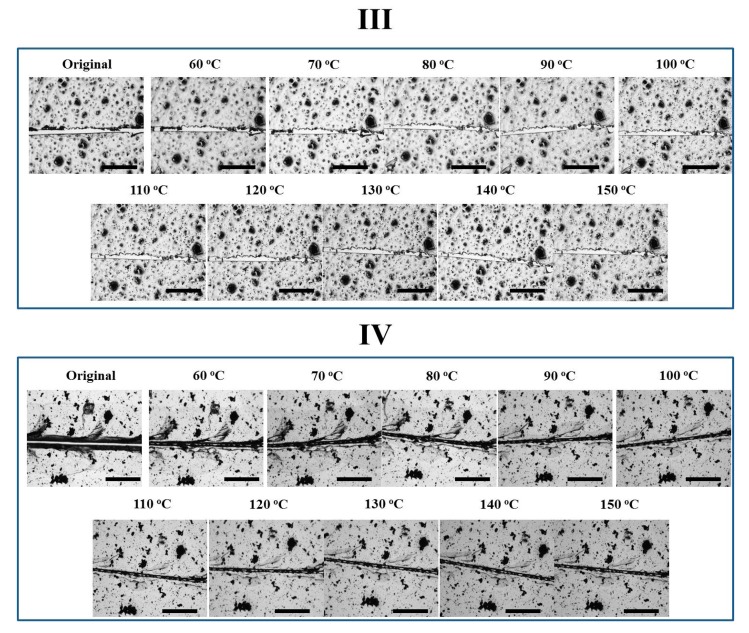
Optical images of the coatings with Networks III–IV under thermal treatment of different temperatures for 10 min each. The scale bar stands for 300 μm.

The four coatings were first treated through a stepwise heating strategy (Figure 4 and Figure 5). The crack width in Network I decreased from 30 to 0 μm at 60 and 150 °C, indicating a reversible plasticity-driven gap closure (Figure 4) [23,24]. The force damaging the coating caused the localized plastic deformation and temporary chain orientation in the areas close to crack surfaces. Upon heating, with the aid of irreversible netpoints, the oriented chains rebounded to their relaxed status and resulted in the observable gap closure. Since physical diffusion and re-entanglement of polymer chains was not allowed for such dense network, the crack was still visible at a high temperature. The second repair step, thus, did not occur efficiently. Also driven by reversible plasticity, Network II presented a gap closure from 20 to 5 μm with increasing the temperature from room temperature to 90 °C (Figure 4). However, the gap became wider again from 5 to 30 μm with further increasing the temperature from 100 to 150 °C. Initiating from 80 °C, the rDA reaction resulted in the de-crosslinking of the reversible network, eliminating the reversible plasticity and correspondingly preventing the sufficient contact of crack surfaces. The “released” fractured Oligomer I with free mobility (*T* > *T*_g_ of 37 °C) may dewet the underlying glass substrate, leading to an even wider gap than the previous one.

As far as Network III was concerned, the gap did not vary evidently with temperature (Figure 5). A low crosslink density possibly may impair the reversible plasticity, i.e., upon heating, the temporary chain orientation relaxed locally instead of “rebounding back”. Besides, since the DGEBA-FA oligomer tended to de-wet the underlying substrate, the gap closure was not favored by surface tension driven viscoelastic flow on glass plates either. Meanwhile, the presence of the irreversible network may prevent the occurrence of dewetting, anchoring the coating towards the substrate with increasing temperature. Finally, the crack in Network IV closed till 90 °C but varied negligibly later. Even though Network IV exhibited a lower crosslinking density than Network III, the gap closure still took place. We crudely speculate that the reversible plasticity was dominated by both crosslinking density and orientation level. The lower crosslinking density in Network IV may result in a high orientation during deformation and correspondingly the high tendency of rebounding, since crosslinking limited the chain mobility and accordingly the degree of molecular orientation during deformation [37]. After the disappearance of the reversible network at a high temperature, the irreversible coupling may also retard the coating dewetting. Therefore, the gap maintained its narrow status, instead of becoming wider again as behaved by Network II.

**Figure 6:**
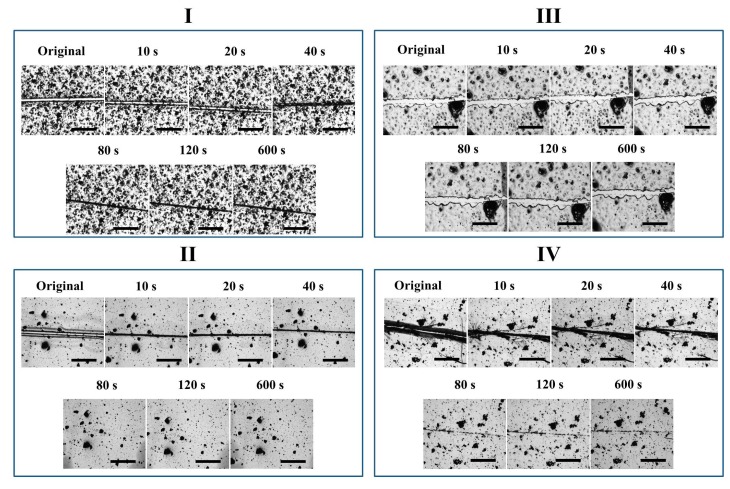
Optical images of how the cracks on the four coatings varied at 150 °C. The scale bar stands for 300 μm.

The four coatings were also directly treated at 150 °C to observe their self-healing performances. As shown in Figure 6, the crack on Network I narrowed down very quickly within 40 s driven by reversible plasticity, while the crack was observed all the time. Network III did not exhibit evident variation in crack width, which is similar to the case in stepwise heating. In comparison, upon direct heating at 150 °C, the self-healing behaviors of Networks II and IV with reversible bonds were different. Network II presented a complete gap closure in 40 s, facilitating the second repair step and causing a crack disappearance after only 80 s. Direct heating at a high temperature triggered an immediate gap closure by reverse plasticity, while the tight contact of crack surfaces allowed Oligomer I resulting from rDA reaction to diffuse into the opposite crack surface, preventing the occurrence of dewetting and ensuring the complete crack repair. Based on an image analysis method as described previously [32], the healing efficiency was calculated to be 91 ± 6%, suggesting a sufficient crack repair. Similarly, Network IV also exhibited a gap closure in 40 s, while the crack to some extent disappeared after 80 s. Since less reversible bonds were incorporated, the complete crack repair was not achieved. In addition, the post-curing may exist in Network IV treated at 150 °C. After the de-coupling of the reversible crosslinking points, the unreacted epoxy groups could be further cured to limit the healing procedures.

Based on the results above, the coating with Network II presented the best thermally-induced self-healing capability on glass plates only in the case of direct heating at 150 °C. The influence of network architecture on self-healing behaviors can, to some extent, be discussed. The crack closure can be contributed by reversible plasticity [23,24] or surface-tension-driven flow [12], while the repair can be achieved by physical molecular re-entanglement [9], reversible non-covalent reactions [38], or reversible covalent reactions in this case. Dense networks (I and II) with a high crosslinking density usually facilitate gap closure driven by reversible plasticity. The disadvantages of the dense irreversible network in complete crack repair has been improved by mixing electrospun thermoplastic poly(*ε*-caprolactone) fibers into epoxy coatings to ensure further gap filling [24]. The dense reversible network enabled not only the gap closure, but complete crack repair as well, leading to complete crack disappearance. The loose network or linear architecture is required for surface-tension-driven flow. We reported another two epoxy coatings with the loose irreversible network, which presented the gap closure and crack disappearance due to chain diffusion and re-entanglement [9,32]. However, in the case here, the surface tension did not drive the gap closure but triggered the dewetting, leading to the failure of self-healing. Therefore, the loose irreversible network cannot guarantee good self-healing. Furthermore, sufficient reversible plasticity-driven gap closure must be triggered before the de-crosslinked oligomers in reversible networks which finally contributed to the chain diffusion and further repair due to regeneration of reversible crosslinks.

### 3.4. Properties and Anti-Corrosion Behaviors of the Four Coatings

In addition to the investigations on structures, thermo-reversibility, and self-healing performance, there is a need to evaluate the practical properties of the coating with Network II. Therefore, the basic properties and anti-corrosion performance of Network II were investigated, in addition to the other three networks, to obtain a global view. The film thickness of the four coatings was 26 ± 1 μm.

**Figure 7:**
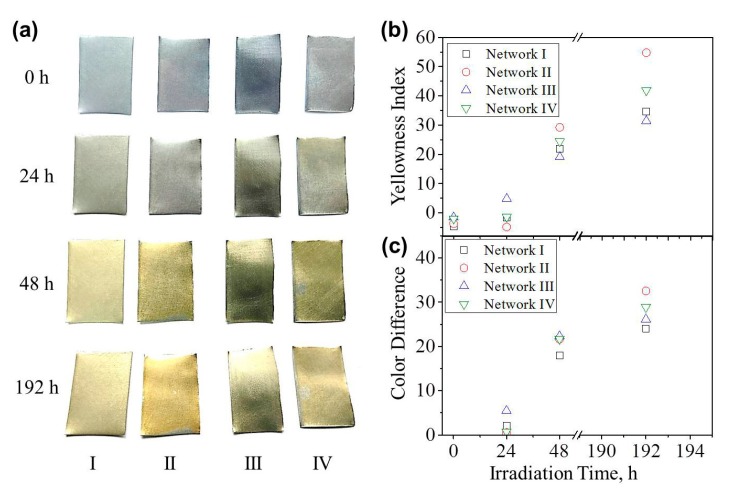
(**a**) Photos of the coatings with Networks I–IV after UV illumination; (**b**,**c**) Correspondingly calculated (**b**) yellowness index and (**c**) color difference.

As shown in Figure 7a, an evident color variation in all four coatings occurred after UV illumination for 48 h. Further increasing the irradiation time deepened the yellow color more slowly. The formed chromophoric impurities generated a protective layer on the coating surface and prevented further degradation. The corresponding yellowness index and the color difference from the as-prepared coating were calculated and are shown in Figure 7b,c. It has been reported that the amine structures were dominant in color development [39]. In our case, Network II presented the largest yellowness index and color variation, suggesting the worst UV aging resistance.

Surface hardness, adhesion, and flexibility are three significant mechanical properties for polymer coatings. Hardness can be used to evaluate the capability of withstanding a certain amount of physical damage, and sufficient hardness obviously reduces the demand of self-healing. The degree of adhesion determines the interaction force between a coating and the underlying substrate, and good adhesion ensures that the damage cannot result in secondary fracture, and facilitates healing. Flexibility is a critical property to assess whether the coating maintains its steady shape when the underlying substrate is bent. As shown in Table 1, Network I exhibited the highest pencil hardness of 3H. Because of the reduced *T*_g_ and crosslinking density, the coating hardness of Network II decreased to 1H–2H, which further reduced to HB–1H for Network IV. It is a conflict that self-healing and surface hardness have inverse requirements for chain mobility. In comparison with the self-healing mechanism based on chain diffusion and re-entanglement, the reversible crosslinking network retarded hardness deterioration. Besides, the adhesion level was improved to some extent from Grade 2–3 in Network I to Grade 1–2 in Networks II, III, and IV, while the flexibility did not vary with the coating networks. In comparison with the conventional epoxy coating with a dense irreversible network, the coating with the self-healing capability based on a reversible DA reaction did not show the evident deterioration in the mechanical properties.

**Figure 8:**
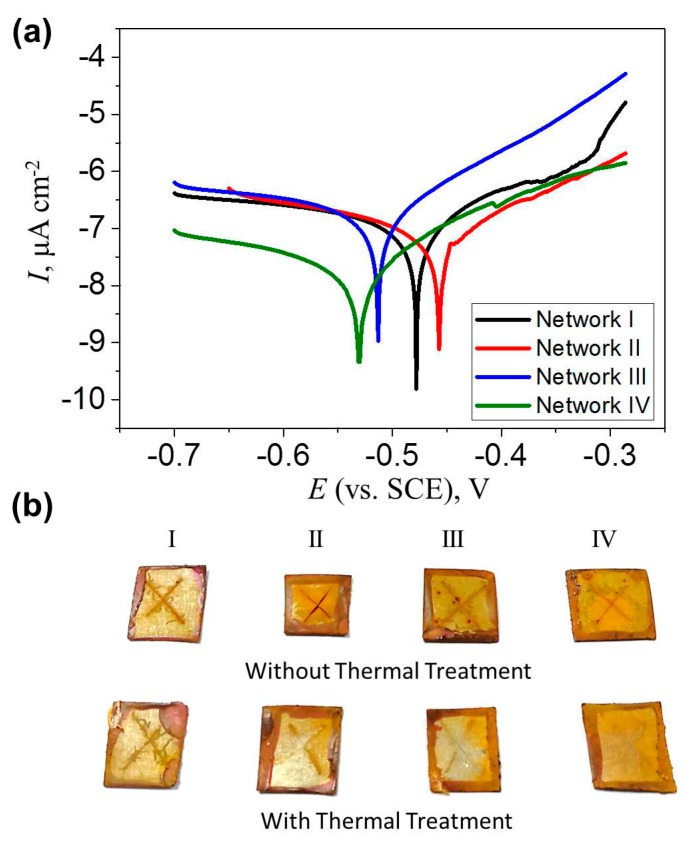
(**a**) Tafel plots of Networks I–IV; (**b**) Photos of the coated steel plates without and with thermal treatment after immersion in NaCl solution for 120 h.

Figure 8a shows the Tafel plot of epoxy coatings with four different network architectures to evaluate their electrochemical corrosion properties. The hyperbolic plot of Network I shifted towards a more positive corrosion potential (*E*_corr_) as reversible Network II was involved. The corrosion current (*I*_corr_), which was obtain from the Tafel plot via extending the straight lines along the linear portions of anodic and cathodic plots as well as extrapolating it to the *E*_corr_ axis, decreased slightly. Polarization resistance (*R*_p_) is given using the Stern–Geary equation, [40]

(2) $$R_{p}=\frac{\beta_{a}\beta_{c}}{2.303\left(\beta_{a}+\beta_{c}\right)I_{\mathrm{corr}}}$$

where, *β*_a_ and *β*_c_ are the anodic and cathodic Tafel constants. As shown in Table 1, the *R*_p_ increased from 5.66 to 10.89 MΩ cm^−2^ when the irreversible network was replaced by the reversible network. The increased *E*_corr_ and *R*_p_, as well as the reduced *I*_corr_ indicated that Network II offered slightly better corrosion protection on tinplates. In comparison, Networks III and IV exhibited weakened corrosion protection, as shown in Figure 8a and Table 1. The corrosion process in a neutral chloride solution consists of an anodic process where Fe becomes Fe^2+^, and a cathodic process where OH– is generated by O_2_ and H_2_O. Fe_2_O_3_ finally generates on a metal surface via chemical oxidation processes. The existence of a polymer coating retards the diffusion of H_2_O and O_2_ onto the metal surface and inhibits metal oxidation. Here, due to its high crosslinking density, Network I presented a good anti-corrosion property, which was slightly improved in Network II with a lower crosslinking density. The reason was possibly related to the solvent difference in evaluating the crosslinking level (DMF) and the anti-corrosion property (water). Therefore, the water uptake ratio of the four networks was measured as the mass increase ratio of the sample immersed in water for 7 days, i.e., the swelling ratio in water. As shown in Table 1, since the water uptake ratio of Network II can be comparable with that of Network I, both of them effectively retarded the corrosive media to pass through the coatings. The slight reduction in adhesion might lead to the easy generation of a metal oxidation layer on Network I. Because of the high water uptake ratios, the anti-corrosion property was weakened in Networks III and IV. In addition, the four networks exhibited the similar hydrophilicity, as determined by the close contact angle values in Table 1, which influenced negligibly the anti-corrosion property.

Finally, the self-healing behaviors of the four coatings were assessed by their corrosion protection performance. The damaged coatings were heated at 150 °C for 10 min before immersing in 3.5 wt % NaCl solution. It can be seen in Figure 8b that after 120 h immersion, severe corrosion occurred in the four cracked coatings without any healing procedures. After the thermal treatment, the coating with a dense irreversible Network I exhibited a similar rust situation as the one with the fresh crack. On the contrary, the other three healed coatings presented less rust on the surfaces. The undamaged coating prevents the contact between the NaCl solution and the underlying steel plates. Therefore, the gap closure presented by Network I still allowed the penetration of the corrosive medium onto the substrate, while the improvement in the corrosion resistance indicated the efficient self-healing of the cracks on the coatings with Networks II–IV. Interestingly, the coating with Network III presented good healing efficiency as well, indicating that the surface tension on steel plates may drive the viscoelastic flow of the materials before the ultimate healing of the cracks.

## 4. Conclusions

Epoxy coatings with four network architectures were prepared to examine how the gap closure and crack repair steps occurred within the thermally-induced self-healing behavior, and to evaluate their practical properties. Only the coating with a dense irreversible Network I with the highest *T*_g_ and crosslinking density presented gap closure upon heating. The crack was still clearly visible at the high temperature. The dense reversible Network II was prepared based on the Diels–Alder reaction, while its thermal reversibility was demonstrated by FTIR, DSC, and swelling experiments. The crack on the coating with a dense reversible network did not close and disappear upon stepwise heating, while the direct heating at 150 °C resulted in the quick gap closure in 40 s and the crack vanishment in 80 s. The crack on the coating with the loose irreversible Network III did not perform evident variation. The heating at a high temperature finally caused partial crack disappearance for the partially irreversible and partially reversible Network IV.

The hardness, adhesion, and flexibility of the four coatings followed the orders as I > II > III > IV, I < II = III = IV, and I = II = III = IV, respectively. Network II showed the best electrochemical corrosion property, even though it exhibited the greatest yellowness development following UV light exposure. Besides, the coatings with Networks II–IV presented better healing efficiency of the anti-corrosion property than Network I.

Upon direct heating to a high temperature, the coating with a dense reversible network based on a DA reaction presented quick gap closure and crack disappearance, indicating good self-healing efficiency. More importantly, the basic properties of the coating did not deteriorate in comparison with the conventional coating with a dense irreversible network, ensuring its further exploration. Therefore, further work may involve DA-reversible bonds in other polymer coating systems, especially high-performance coatings.

## Figures and Tables

**Table 1:** Thermal, mechanical, and electrochemical corrosion properties of the four coatings.

Properties	Thermo-Degradation Property			Mechanical Property			Electrochemical Corrosion			Water Uptake Ratio	Contact Angle
Netwoks	*T*_g_	*T*_d, 5%_	*T*_d, 50%_	Pencil Hardness	Flexibility	Adhesion	*E*_corr_	*I*_corr_	*R*_p_	--	--
	°C	°C	°C	N/A	mm	N/A	mV	μA cm^−2^	MΩ cm^−2^	%	°
I	118	305	429	2H-3H	2	2-3	−477	0.149	5.66	1.2	79
II	69	164	462	1H-2H	2	1-2	−457	0.106	10.89	4.8	76
III	33	144	383	1H-2H	2	1-2	−512	0.182	5.50	30.9	73
IV	N/A	160	400	HB-1H	2	1-2	−530	0.238	5.16	30.3	74
